# The Impact of COVID-19 Surges in 2019–2021 on Patient-Reported Outcome Measures After Spine Surgery at an Academic Tertiary Referral Center in Taiwan: A Retrospective Observational Cohort Study

**DOI:** 10.3389/fsurg.2022.853441

**Published:** 2022-03-17

**Authors:** Yu-Hsien Lin, Jun-Sing Wang, Wen-Chien Wang, Yu-Tsung Lin, Yun-Che Wu, Kun-Hui Chen, Chien-Chou Pan, Ning-Chien Chin, Cheng-Min Shih, Cheng-Hung Lee

**Affiliations:** ^1^Department of Orthopedics, Taichung Veterans General Hospital, Taichung, Taiwan; ^2^Department of Medicine, School of Medicine, National Yang Ming Chiao Tung University, Taipei, Taiwan; ^3^Division of Endocrinology and Metabolism, Department of Internal Medicine, Taichung Veterans General Hospital, Taichung, Taiwan; ^4^Ph.D. Program in Translational Medicine, National Chung Hsing University, Taichung, Taiwan; ^5^College of Medicine, National Chung Hsing University, Taichung, Taiwan; ^6^Department of Computer Science and Information Engineering, Providence University, Taichung, Taiwan; ^7^Department of Rehabilitation Science, Jenteh Junior College of Medicine, Nursing and Management, Miaoli, Taiwan; ^8^Department of Physical Therapy, Hung Kuang University, Taichung, Taiwan; ^9^Department of Food Science and Technology, Hung Kuang University, Taichung, Taiwan

**Keywords:** coronavirus disease 2019, health care quality, pandemic, patient report outcome, spine surgery

## Abstract

**Aim:**

Limited data are available on the impact of the coronavirus disease 2019 (COVID-19) pandemic on patient-reported outcome measures (PROMs) in patients who underwent spine surgery. In this study, we aimed to investigate the associations between the COVID-19 outbreak in Taiwan (May 2021) and PROMs in patients who underwent spine surgery.

**Method:**

We retrospectively identified patients who underwent spine surgery during identical defined 6-week time-intervals (May 16 to June 30) in 2019, 2020, and 2021. PROMs, including visual analog scale (VAS) score for pain, Oswestry disability index (ODI), and EuroQol-5D (EQ-5D), were investigated before surgical intervention and at a 1-month follow-up. Relevant clinical information was collected from the electronic medical records of patients. Linear regression analysis was used to examine the association between the pandemic in 2021 (vs. 2019/2020) and the PROMs after adjusting for age, sex, and relevant clinical variables.

**Results:**

The number of patients who underwent spine surgery at our hospital during the identical defined 6-week time-intervals in 2019, 2020, and 2021 was 77, 70, and 48, respectively. The surgical intervention significantly improved VAS, ODI, and EQ-5D of the patients (1 month after surgery vs. before surgery, all *p* < 0.001) in all three study periods. However, there was a significant between-group difference in change from baseline in VAS (*p* = 0.002) and EQ-5D (*p* = 0.010). The decrease in VAS and increase in EQ-5D after surgery in 2021 were not as much as those in 2019 and 2020. The associations between the pandemic in 2021 (vs. 2019/2020) and changes in VAS (β coefficient 1.239; 95% confidence interval [CI] 0.355 to 2.124; *p* = 0.006) and EQ-5D (β coefficient, −0.095; 95% CI, −0.155 to −0.035; *p* = 0.002) after spine surgery were independent of relevant clinical factors.

**Conclusion:**

There was less improvement in short-term PROMs (VAS and EQ-5D) after spine surgery during the COVID-19 pandemic. Assessment of PROMs in surgical patients during a pandemic may be clinically relevant, and psychological support in this condition might help improve patients' outcomes.

## Introduction

Since the coronavirus disease 2019 (COVID-19) pandemic (1), the health care system worldwide has encountered a huge challenge. The large patient volume, shortage of medical staff, and imperative quarantine and lockdown changed daily medical practice and health care quality (2, 3). For example, an increase in body weight and body mass index during the pandemic may be associated with an increased incidence of cardiovascular risk factors and subsequent risk of cardiovascular diseases (4, 5).

Similar conditions have also been seen in surgeries (6). The impact of the pandemic on surgical volume may lead to postponement of elective surgeries (7–9), which may have unfavorable effects on patient outcomes (10). In fact, a recent global survey (11) showed that hip fracture service was disrupted during the pandemic, thereby impacting health and social care experienced by the patients. This dilemma was also noted in cardiovascular (12), gastrointestinal, and (13) oncology (14) surgeries.

Moreover, the aforementioned issues may have negative effects on patients' psychosocial function (15). Patient-reported outcome measures (PROMs) are important assessment of quality in spine surgery (16). However, there are limited data on the impact of the COVID-19 pandemic on PROMs in patients who underwent spine surgery. Furthermore, a COVID-19 outbreak occurred in Taiwan in May 2021. This study aimed to investigate the associations between the COVID-19 outbreak in Taiwan and PROMs in patients who underwent spine surgery.

## Methods

We hypothesized that a pandemic may have an impact on PROMs in patients undergoing spine surgery. A level 3 outbreak alert for COVID-19 was announced by the Taiwan Center of Disease Control on May 15, 2021. We conducted a retrospective study to investigate the changes in PROMs after spine surgery before and after the level 3 outbreak in Taiwan. A total of 13,329 patients had confirmed COVID-19 in Taiwan between May 16, 2021, and June 30, 2021 (only 7 patients had confirmed COVID-19 during the same time frame in 2020). Hence, we retrospectively identified patients who underwent spine surgery in our hospital during identical defined 6-week time-intervals (May 16 to June 30) in 2019, 2020, and 2021. Several measures of perioperative and postoperative quality of care, as well as PROMs in the three study periods, were investigated. This study was conducted following the Declaration of Helsinki. The study protocol was approved by the Institutional Review Board of Taichung Veterans General Hospital, Taichung, Taiwan (approval number: CE21395B).

We included adult patients who were admitted for spine surgery during the study period. Several PROMs, including visual analog scale (VAS) for pain (17), Oswestry disability index (ODI) (18), and EuroQol-5D (EQ-5D) (19), were investigated before surgical intervention and at 1-month follow-up. Patients who had missing information on the PORMs were excluded. Assessment of PROMs was conducted by a trained nurse as part of pre-operative evaluation. The assessment was conducted again 1 month after the surgery at outpatient clinic or by phone calls. A higher VAS or ODI score indicates greater severity of pain and disability, respectively. The EQ-5D consists of assessment for health states in five dimensions (16)—mobility, self-care, usual activities, pain/discomfort, and anxiety/depression. The original scores of the five dimensions were transformed to a final score. A higher value indicates a better quality of life.

Relevant clinical information was collected from the electronic medical records of patients. The indication for surgery was clarified and classified as trauma of spine, degeneration/deformity of spine, and infection/neoplasm/others. The followings were considered as an emergency diagnosis: compression fracture, burst fracture/fracture-dislocation, tumor/metastasis, cauda equina syndrome/myelopathy/motor weakness, and infection. The level of spine surgery, presence of spinal cord injury, type of procedure, duration of surgery, intraoperative blood loss, neurological complications, and unplanned surgical revision were recorded. Measures of perioperative quality of care included waiting days from outpatient clinic to admission for surgery, waiting hours from the emergency department visit to the surgery, and length of hospital stay. Measures of postoperative quality of care included outpatient clinic follow-up rate and emergency department visit within 3 days or readmission within 14 days after hospital discharge.

### Statistical Analysis

Statistical analyses were performed using the Statistical Package for the Social Sciences (IBM SPSS version 22.0; International Business Machines Corp, NY, USA). Changes from baseline to 1-month follow-up in the PROMs were compared using the Wilcoxon signed-rank test. Statistical differences in the variables across the three study time frames were examined using the Kruskal–Wallis test. The associations between the pandemic in 2021 (vs. 2019/2020) and the PROMs after adjustment for age, sex, and relevant clinical variables were examined using linear regression analysis. A *p-*value of <0.05 was considered statistically significant in all analyses.

## Results

The number of patients who underwent spine surgery at our hospital during the identical defined 6-week time-intervals in 2019, 2020, and 2021 was 77, 70, and 48, respectively (Table 1). There was a trend toward a decrease in surgical volume in 2021 (6.9 ± 2.7 operations per week), compared to that in 2019 (11.0 ± 2.6 operations per week) and 2020 (10.0 ± 5.0 operations per week) (*p* = 0.069). A higher proportion of patients who underwent spine surgery in 2021 had diabetes (37.5%) compared to the number of patients who underwent spine surgery in 2019 (16.9%) and 2020 (15.7%) (*p* = 0.008). There were no significant between-group differences in the other variables (Table 1). Surgical procedures and complications are presented in Table 2. There were no significant between-group differences in these variables.

**Table 1:** Characteristics of the study population according to the year of surgery.

**Variables**	**2019 (May 16–June 30)**	**2020 (May 16–June 30)**	**2021 (May 16–June 30)**	***p*-value**
Number of patients	77	70	48	
Number of operations per week	11.0 ± 2.6	10.0 ± 5.0	6.9 ± 2.7	0.069
Age, years	70.0 (58.5, 76.0)	68.0 (55.0, 78.5)	66.0 (52.3, 74.0)	0.290
Male, *n* (%)	30 (39.0)	34 (48.6)	19 (39.6)	0.446
Body mass index, kg/m^2^	24.3 (21.0, 27.5)	25.3 (22.8, 27.8)	26.1 (23.7, 29.9)	0.095
Diabetes, *n* (%)	13 (16.9)	11 (15.7)	18 (37.5)	0.008
Hypertension, *n* (%)	19 (24.7)	17 (24.3)	18 (37.5)	0.216
Admission identity, *n* (%)				0.113
Via emergency department	7 (9.1)	15 (21.4)	8 (16.7)	
Via outpatient department	70 (90.9)	55 (78.6)	40 (83.3)	
With an emergent diagnosis, *n* (%)	39 (50.6)	35 (50.0)	25 (52.1)	0.975

*Values are presented as median (interquartile range) or n (%)*.

**Table 2:** Surgical procedures and complications of the study population according to the year of surgery.

**Variables**	**2019 (May 16–June 30)**	**2020 (May 16–June 30)**	**2021 (May 16–June 30)**	***p*-value**
**Surgical indication**, ***n*** **(%)**				0.429
Trauma of spine	23 (29.9)	26 (37.1)	10 (20.8)	
Compression fracture	15	16	7	
Burst fracture	8	10	3	
Degeneration/deformity of spine	42 (54.5)	36 (51.4)	30 (62.5)	
Infection/neoplasm/others	12 (15.6)	8 (11.4)	8 (16.7)	
Spinal cord injury due to trauma, *n* (%)	2 (2.6)	3 (4.3)	0 (0)	0.466
Level of spine surgery, *n* (%)				0.544
C-spine	2 (2.6)	3 (4.3)	4 (8.3)	
T-spine	17 (22.1)	12 (17.1)	9 (18.8)	
L-S spine	58 (75.3)	55 (78.6)	35 (72.9)	
Type of procedure, *n* (%)				0.249
Thoracolumbar fusion/deformity correction	34 (44.2)	36 (51.4)	22 (45.8)	
Vertebroplasty/Kyphoplasty	18 (23.4)	20 (28.6)	8 (16.7)	
C-spine anterior surgery	2 (2.6)	2 (2.9)	3 (6.3)	
Decompression/discectomy	19 (24.7)	12 (17.1)	12 (25.0)	
Spine tumor	4 (5.2)	0 (0)	3 (6.3)	
Duration of surgery, min	210 (60, 360)	255 (60, 457)	240 (120, 352.5)	0.464
Intraoperative blood loss, ml	100 (0, 425)	200 (0, 800)	200 (0, 737.5)	0.587
Neurological complications, *n* (%)	2 (2.6)	3 (4.3)	1 (2.1)	0.746
Unplanned surgical revision, *n* (%)	3 (3.9)	3 (4.3)	3 (6.3)	0.820

*Values are presented as median (interquartile range) or n (%)*.

Parameters related to the quality of care are summarized in Table 3. Across the three study time frames, there were no significant differences in the time from outpatient/emergency department visit to admission/surgery and length of hospital stay. The surgical intervention significantly improved VAS, ODI, and EQ-5D (1 month after surgery vs. before surgery, all *p* < 0.001) in 2019, 2020, and 2021. Nevertheless, there was a significant between-group difference in change from baseline in VAS (*p* = 0.002) and EQ-5D (*p* = 0.010). The decrease in VAS and increase in EQ-5D after surgery in 2021 were not as much as those in 2019 and 2020. The time interval between hospital discharge and outpatient clinic follow-up was longer in 2021, compared with that in 2019 and 2020. However, there were no significant differences in the rate of outpatient clinic follow-up, emergency department visit within 3 days after hospital discharge, and readmission within 14 days after hospital discharge between the three study time frames (Table 3).

**Table 3:** Parameters related to quality of care according to the year of surgery.

**Variables**	**2019 (May 16–June 30)**	**2020 (May 16–June 30)**	**2021 (May 16–June 30)**	***p*-value**
Time from outpatient clinic visit to admission, days	8.0 (3.0, 18.0)	7.0 (3.0, 21.0)	7.0 (4.0, 15.0)	0.902
Time from emergency department visit to surgery, hours	29.0 (23.0, 77.0)	57.0 (47.0, 103.0)	36.3 (24.5, 62.2)	0.227
Length of hospital stay, days	5.5 (2.0, 9.0)	6.0 (3.0, 10.0)	5.0 (2.3, 7.0)	0.337
**VAS for pain**
Before surgery	8.0 (8.0, 10.0)	8.0 (7.5, 10.0)	7.5 (6.0, 8.0)	0.001
One month after surgery	4.0 (3.0, 5.0)*	4.5 (3.0, 6.0)*	3.0 (2.0, 6.0)*	0.585
Change from baseline	−4.0 (−7.0, −3.0)	−4.0 (−5.0, −3.0)	−3.0 (−4.0, −2.0)	0.002
**Oswestry disability index**
Before surgery	62.2 (52.8, 71.1)	62.2 (53.3, 71.1)	63.3 (53.3, 72.8)	0.825
One month after surgery	48.9 (42.2, 61.1)*	50.0 (40.0, 59.5)*	48.9 (36.7, 57.8)*	0.937
Change from baseline	−13.3 (−20.0, −2.2)	−11.1 (−20.0, −2.8)	−13.3 (−20.0, −6.7)	0.526
**EQ-5D**
Before surgery	0.3 (0.3, 0.3)	0.3 (0.3, 0.3)	0.3 (0.2, 0.5)	0.529
One month after surgery	0.6 (0.5, 0.6)*	0.6 (0.5, 0.6)*	0.5 (0.5, 0.6)*	0.117
Change from baseline	0.3 (0.2, 0.3)	0.2 (0.2, 0.3)	0.2 (0.0, 0.2)	0.010
Postoperative outpatient clinic follow-up rate	76 (95.0)	70 (95.9)	48 (100)	0.306
Time from hospital discharge to outpatient clinic follow-up, days	8.0 (6.3, 12.0)	8.0 (7.0, 11.0)	10.5 (8.0, 13.0)	0.006
Emergency department visit within 3 days after hospital discharge	1 (1.3)	0	0	0.468
Readmission within 14 days after hospital discharge	2 (2.5)	2 (2.7)	1 (2.1)	0.975

Values are presented as median (interquartile range) or n (%). ^*^p <0.001 vs. before surgery. EQ-5D, EuroQol-5D; VAS, visual analog scale.

Table 4 shows the original scores of the five dimensions of EQ-5D before and 1 month after surgery in the three study periods. There were no significant between-group differences regarding the changes from baseline to 1-month follow-up in scores for mobility, self-care, and usual activities. The improvement (1 month after surgery vs. before surgery) in pain/discomfort and anxiety/depression was significant (*p* < 0.001) in 2019, 2020, and 2021. Similar to the findings in Table 3, the decrease in pain/discomfort (*p* = 0.001) and anxiety/depression (*p* < 0.001) after surgery in 2021 was not as much as that in 2019 and 2020.

**Table 4:** Original scores of the five dimensions of EQ-5D according to the year of surgery.

**Variables**	**2019 (May 16–June 30)**	**2020 (May 16–June 30)**	**2021 (May 16–June 30)**	***p*-value**
**Mobility**
Before surgery	2.0 (2.0, 2.0)	2.0 (2.0, 2.0)	2.0 (2.0, 2.0)	0.438
One month after surgery	2.0 (2.0, 2.0)*	2.0 (2.0, 2.0)**	2.0 (1.0, 2.0)	0.268
Change from baseline	0 (0, 0)	0 (0, 0)	0 (0, 0)	0.917
**Self-care**
Before surgery	2.0 (2.0, 2.0)	2.0 (2.0, 2.0)	2.0 (2.0, 2.0)	0.020
One month after surgery	2.0 (2.0, 2.0)**	2.0 (2.0, 2.0)	2.0 (2.0, 2.0)	0.031
Change from baseline	0 (0, 0)	0 (0, 0)	0 (0, 0)	0.243
**Usual activities**
Before surgery	2.0 (2.0, 2.0)	2.0 (2.0, 2.0)	2.0 (2.0, 3.0)	0.004
One month after surgery	2.0 (2.0, 2.0)*	2.0 (2.0, 2.0)	2.0 (2.0, 2.0)*	0.040
Change from baseline	0 (0, 0)	0 (0, 0)	0 (0, 0)	0.131
**Pain/discomfort**
Before surgery	3.0 (3.0, 3.0)	3.0 (3.0, 3.0)	3.0 (2.0, 3.0)	0.006
One month after surgery	2.0 (2.0, 2.0)***	2.0 (2.0, 2.0)***	2.0 (2.0, 2.0)***	0.089
Change from baseline	−1.0 (−1.0, −1.0)	−1.0 (−1.0, −1.0)	0 (−1.0, 0)	0.001
**Anxiety/depression**
Before surgery	3.0 (3.0, 3.0)	3.0 (3.0, 3.0)	3.0 (2.0, 3.0)	<0.001
One month after surgery	1.0 (1.0, 2.0)***	2.0 (1.0, 2.0)***	2.0 (1.0, 2.0)***	0.051
Change from baseline	−2.0 (−2.0, −1.0)	−1.0 (−2.0, −1.0)	−1.0 (−1.0, 0)	<0.001

Values are presented as median (interquartile range). ^*^p <0.05, ^**^p <0.01, ^***^p <0.001 vs. before surgery. EQ-5D, EuroQol-5D.

The associations between the pandemic in 2021 (vs. 2019/2020) and changes from baseline in VAS and EQ-5D after spine surgery are shown in Table 5. There was a positive association between surgery in 2021 (vs. 2019/2020) and change from baseline in VAS (β coefficient 1.563; 95% confidence interval [CI] 0.700 to 2.427; *p* < 0.001; Model 1). The association remained significant (β coefficient, 1.239; 95% CI, 0.355 to 2.124; *p* = 0.006; Model 4) after adjustment for age, sex, and relevant clinical variables. Similarly, there was a negative association between surgery in 2021 (vs. 2019/2020) and change from baseline in EQ-5D (β coefficient −0.086; 95% CI −0.147 to −0.025; *p* = 0.006; Model 1). The association was independent of other relevant clinical variables (β coefficient, −0.095; 95% CI, −0.155 to −0.035; *p* = 0.002; Model 4).

**Table 5:** Associations between the COVID-19 pandemic in 2021 and changes from baseline to 1-month follow-up in VAS and EQ-5D scores.

	**Change in VAS score from baseline**		**Change in EQ-5D score from baseline**	
**2021 vs. 2019/2020**	**β coefficient (95% CI)**	***p*-value**	**β coefficient (95% CI)**	***p*-value**
Model 1	1.563 (0.700, 2.427)	<0.001	−0.086 (−0.147, −0.025)	0.006
Model 2	1.527 (0.670, 2.384)	0.001	−0.088 (−0.150, −0.026)	0.006
Model 3	1.208 (0.324, 2.091)	0.008	−0.086 (−0.149, −0.023)	0.008
Model 4	1.239 (0.355, 2.124)	0.006	−0.095 (−0.155, −0.035)	0.002

*Model 1, unadjusted. Model 2, adjusted for age and sex. Model 3, adjusted for variables in Model 2 plus body mass index and history of diabetes. Model 4, adjusted for variables in Model 3 plus admission identity (via emergency department or outpatient department) and whether the patient had an emergency diagnosis. CI, confidence interval; COVID-19, coronavirus disease 2019; EQ-5D, EuroQol-5D; VAS, visual analog scale*.

## Discussion

In this study, we reported that the decrease in VAS and increase in EQ-5D after spine surgery in 2021 were not as much as those in 2019 and 2020. We demonstrated independent associations between spinal surgery in 2021 (vs. 2019/2020) and changes in VAS and EQ-5D in the COVID-19 pandemic period in Taiwan. To the best of our knowledge, this is the first study to investigate the association between the COVID-19 pandemic and PROMs after spine surgery.

Clinical practices have changed since 2020 and affected numerous branches of medicine such as surgery (20). Studies have reported a decrease in surgical volume and the postponement of surgical procedures (21). These factors may increase psychological stress in surgical patients during the pandemic period (22–24). Moreover, psychological stress may affect PROMs after orthopedic surgery (25–27). These findings may explain why patients showed less improvement in VAS and EQ-5D after spinal surgery during the pandemic (Table 3). The associations between the pandemic in Taiwan (2021 vs. 2019/2020) and PROMs were independent of several confounders (Table 5). This was further supported by the finding that patients showed less improvement in the dimension of anxiety/depression during the pandemic (Table 4).

There were no significant differences in the waiting time from outpatient clinic visit to admission for surgery, the time from emergency department visit to the surgery, and length of hospital stay across the three study periods (Table 3). Despite the prolonged time from hospital discharge to outpatient clinic follow-up, there were no significant differences in terms of the postoperative outpatient clinic follow-up rate and unscheduled emergency department visit within 3 days or readmission within 14 days after hospital discharge. A higher proportion of our patients in 2021 had diabetes (Table 1). This finding might be explained by that patients with diabetes were considered as a priority for surgery when the surgical volume was affected by the pandemic. None of the patients had COVID-19 during the study period. These findings revealed the efforts undertaken to support the quality of surgical care during the pandemic. Minor surgery was considered safe during the COVID-19 pandemic in a recent report (28). Although half of the patients in this study had an emergency diagnosis (Table 1), the surgical procedures were safely conducted during the pandemic with objective measures of quality of care similar to those in the previous 2 years.

Since limited studies have investigated the effect of the pandemic on PROMs of surgical procedures, our findings are clinically relevant as important PROMs associated with surgical complications (16, 29, 30) are occasionally overlooked in clinical practice. The significant associations between spinal surgery during the pandemic period and changes in PROMs (VAS and EQ-5D) highlight the importance of psychological support in medical and surgical care during the COVID-19 period (31, 32).

There are several limitations to this study. First, this was a retrospective study conducted at a single center with relatively small sample size. Further large-scale prospective studies in patients who underwent orthopedic surgery are needed to validate the findings of this study. Second, the PROMs were assessed before and 1 month after surgery. The effects of a pandemic on long-term outcomes in patients who underwent spine surgery warrant further investigation. With these limitations in mind, the findings provided insights on surgical care for patients who underwent spine surgery during a pandemic.

## Conclusion

We demonstrated less improvement in short-term PROMs (VAS and EQ-5D) after spine surgery during the pandemic in Taiwan. The associations between the pandemic and improvements in PROMs were independent of several confounders. Assessment of PROMs in surgical patients during a pandemic may be clinically relevant, and psychological support in this condition might help improve patients' outcomes. Further studies are needed to confirm these findings.

## Data Availability Statement

The original contributions presented in the study are included in the article/supplementary material, further inquiries can be directed to the corresponding author/s.

## Author Contributions

Y-HL, J-SW, and C-HL designed and conducted the research. Y-HL, W-CW, Y-TL, Y-CW, K-HC, C-CP, N-CC, and C-MS contributed acquisition of data, analysis, and interpretation of data. Y-HL, J-SW, and W-CW wrote the first draft of the manuscript. Y-TL, Y-CW, K-HC, C-CP, N-CC, C-MS, and C-HL revised the manuscript critically for important intellectual content. All authors approved the final draft of the manuscript.

## Conflict of Interest

The authors declare that the research was conducted in the absence of any commercial or financial relationships that could be construed as a potential conflict of interest.

## Publisher's Note

All claims expressed in this article are solely those of the authors and do not necessarily represent those of their affiliated organizations, or those of the publisher, the editors and the reviewers. Any product that may be evaluated in this article, or claim that may be made by its manufacturer, is not guaranteed or endorsed by the publisher.
